# Psychological Distress in Women with Fibromyalgia: The Roles of Body Appreciation, Self-Compassion, and Self-Criticism

**DOI:** 10.1007/s12529-024-10302-5

**Published:** 2024-06-17

**Authors:** Sigal Levy, Shay Ohayon, Ronit Avitsur, Shulamit Geller

**Affiliations:** 1 Statistical Education Unit, The Academic College of Tel Aviv-Yaffo, Tel-Aviv, Israel; 2 School of Behavioral Sciences, The Academic College of Tel Aviv-Yaffo, 14 Rabenu Yeruham St, 68182 Tel-Aviv, Israel

**Keywords:** Fibromyalgia, Body appreciation, Self-criticism, Self-compassion, Psychological distress

## Abstract

**Background:**

While past research detected a direct link between symptoms of fibromyalgia (FM) and psychological distress, body appreciation was suggested as a viable mediator of this link. The aim of the present study was to further develop an explanatory model for the effect of FM on women’s psychological distress and identify possible protective and risk factors. Specifically, it was hypothesized that self-compassion would moderate the indirect effect of body appreciation and self-criticism on psychological distress in women with FM.

**Method:**

This study comprised a total of 293 women, aged 20–68 (M = 34.8, SD = 12.3), of whom 146 were women with FM and 147 were heathy controls. All the women completed questionnaires regarding demographic characteristics, depression (PHQ-9), anxiety (GAD-7), self-criticism (DEQ-SC), body appreciation (BAS2), and the self-compassion scale (SCS).

**Results:**

A moderated serial mediation model demonstrated lower body appreciation in participants with FM compared to controls. These lower levels of body appreciation, together with lower levels of self-compassion, were associated with greater self-criticism and, consequently, higher levels of psychological distress.

**Conclusions:**

The results emphasize the role of self-compassion as a protective mechanism against psychological distress among women with FM. Future studies should further investigate the effect of self-compassion-focused interventions on patients with FM.

**Supplementary Information:**

The online version contains supplementary material available at 10.1007/s12529-024-10302-5.

## Introduction

Fibromyalgia (FM) is a rheumatic disorder characterized by continuous pain, poor sleep quality, fatigue, and cognitive difficulties [1]. The estimated prevalence of FM in the general population ranges between 2 and 5% with higher prevalence in women than men [2]. FM is often associated with psychiatric disorders. For example, a previous study reported that 77.3% of participants with FM had symptoms characteristic of Axis I psychiatric disorders, mainly depression and anxiety [3]. Other studies showed participants with FM displaying higher rates of depression than participants with rheumatoid arthritis, neuropathic pain, or healthy control participants (HP) [4]. Similarly, patients with FM reported higher rates of anxiety disorders [5] and more severe anxiety symptoms than neuropathic pain patients and HP [4]. Depression and anxiety in FM patients are usually attributed to a feeling of isolation and of being misunderstood or rejected by peers and society in general. This, together with the intense pain that characterizes the disease, may contribute to the high prevalence of psychological distress among FM patients [6]. However, it should be noted that the directionality of these effects may be inverse and that FM is indeed a result of the adverse psychological state [7].

A further issue which may contribute to distress in FM is body image. Body image is a multidimensional construct that includes self-perceptions and attitudes toward the body [8, 9]. One important structure of body image is body appreciation, which refers to a positive construct of body image and is conceptualized as the unconditional approval and respect of the body [9]. Body image may play an important role in the well-being and psychological distress of people with chronic diseases. For example, some studies have demonstrated negative correlations between the body image of participants with diseases (e.g., breast cancer, endometriosis) and levels of depression, anxiety, and stress [10, 11]. Studies related to the association between body image of participants with FM is lacking. Few studies have demonstrated that FM patients demonstrated disturbed body image compared to HP [12, 13]. Furthermore, a significant positive correlation was found between level of pain and body image dissatisfaction, among FM patients [12]. It was suggested that body image may be influenced by the illness-affected body parts (especially painful and stiff areas), problems in cognitive function, negative health care experiences, activity limitations, and decreased quality of life [14]. In addition, positive aspects of body image were positively correlated with quality-of-life measures in patients with diseases; higher levels of body appreciation thus predicted better quality of life [15].

Several studies have suggested that the development of psychological distress in association with FM symptoms is mediated, at least partially, by body image. While a few studies found that patients with FM demonstrate lower body appreciation and a distortion in body image perception and functionality [6, 12], our recent study showed that body appreciation mediates the link between FM and psychological distress [16]. In this study, it was demonstrated that body appreciation and social comparison strategies mediated the link between FM and psychological distress [16]. In addition, among FM patients, body appreciation moderated the link between pain intensity and aspects of social comparison strategies [16].

The negative attitudes and concerns about the body experienced by women with FM involve self-perceptions of being negatively viewed and judged as, for example, unattractive or dysfunctional [17] due to their distance from societal body ideals [18, 19]. Often called self-criticism, these self-assessments can be seen as a maladaptive coping mechanism characterized by rigorous self-examination and an overwhelming fear of personal shortcomings [20]. Self-criticism can manifest as clear self-deprecating thoughts or as emotions of guilt, shame, or anger. Both in clinical and general populations, there is a link between self-criticism and symptoms of depression [21]. Self-criticism is described as a continuous dimension that concerns self-appraisal: the positive end of this continuum could lead to self-enhancement, while the negative end could be destructive [22]. The present study investigates the negative aspects of this concept, as it is known to be linked to psychological distress symptoms in patients with chronic conditions [23]. While a recent study has established the relationship between self-critical perfectionism and anxiety levels in participants with FM [24], yet another found that, compared to HP, participants with endometriosis and an additional chronic illness demonstrated lower body image and higher levels of self-criticism which, in turn, predicted higher levels of both anxiety and depression [11]. Similar findings with other chronic health conditions demonstrated that patients with higher levels of self-criticism presented with higher levels of depression [25].

Self-compassion, defined as “non-judgmental understanding of one’s pain, inadequacies, and failures, so that one’s experience is seen as part of the larger human experience” [26], may serve as a natural regulator of mood and feelings of self-worth [27]. It is thus considered an important strategy for coping with negative emotions associated with body image distress [28], self-criticism [29], and depression [30]. In addition, self-compassion was found to have a protective role against the psychological distress associated with lower body image in patients with chronic conditions. For example, in participants with cystic fibrosis, the relationship between body image and anxiety levels was moderated by self-compassion: participants with a more positive body image but lower levels of self-compassion demonstrated higher levels of anxiety than participants with higher levels of self-compassion [31]. Along these lines, a recent study with FM participants has illustrated that self-compassion played a role in the relationships between treatment outcomes and both depression and anxiety [32]. Similarly, it was involved in the effect of social support on mental quality of life among participants with FM [33]. However, the link between self-compassion, body image, self-criticism, and psychological distress among women with FM is not yet fully understood.

**Fig. 1:**
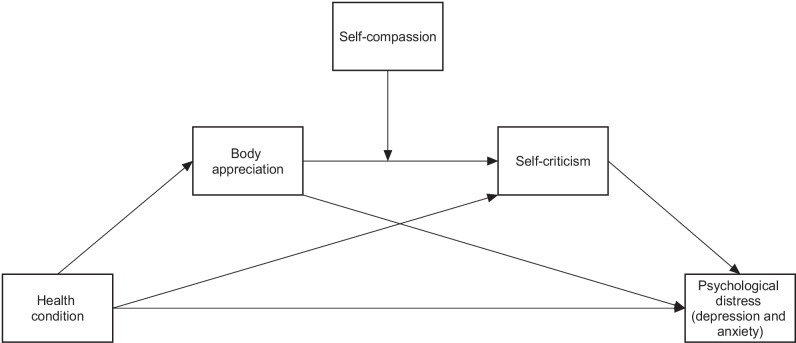
Conceptual model

The overarching goal of the current study is therefore to continue exploring the psychological factors involved in the development of distress associated with FM [16] while identifying the roles and the interplay between body appreciation, self-compassion, and self-criticism in the manifestation of psychological distress in women with FM compared to HP. Specifically, as the theoretical model in Fig. 1 suggests, it was hypothesized that the relationship between health condition (defined as the presence of FM diagnosis) and psychological distress is mediated by both body appreciation and self-criticism, two factors which were previously associated with both FM and psychological distress, compared to HP [12, 24]. It was therefore expected for women with FM to demonstrate lower levels of body appreciation compared to HP, related, in turn, to higher levels of self-criticism followed by psychological distress. In addition, as self-compassion was found to have an important role in treating people with FM [32, 33], and in accordance with previous suggestions regarding the role of self-compassion in the relationship between body appreciation and self-criticism [34, 35], it was hypothesized that the relationship between body appreciation and self-criticism is moderated by self-compassion and thus expected participants with higher levels of self-compassion would demonstrate a weaker relationship between body appreciation and self-criticism.

## Method

### Participants

The data of current study was obtained from a cross-sectional survey which was carried out in Israel during 2020–2021 [16]. Participants were recruited via two different methods: (1) relevant forums over the Internet, including forums that are dedicated to support and coping with FM and other non-specific women forums. Potential volunteers were given a link to a survey and asked to complete it electronically; (2) a snowball/convenience sample—students approached potential participants among their acquaintances, who were, in turn, asked to help expand the sample by recruiting more participants from their social networks in a multi-stage method. Individuals who agreed to take part in the study were sent a link to the survey and asked to complete it electronically. Inclusion criteria were women aged over 20 who were fluent in Hebrew. Participants were asked about any existing medical conditions (FM, high blood pressure, diabetes, heart condition, asthma, irritable bowel disease, and others). The control group was comprised of participants that reported having no existing medical condition, while the FM group was comprised of participants who reported being diagnosed with FM and possibly other diseases. Diagnosis was not corroborated by medical records. The final sample comprised of 293 women, of whom 146 reported being diagnosed with FM and 147 were healthy controls who completed the entire survey. Participants were aged 20–68 (M = 34.3, SD = 12.1).

## Measures

### Body Appreciation

Body appreciation was measured using the Body Appreciation Scale-2 (BAS2) [36]. This is a 10-item measure that assesses acceptance of one’s body, respect and care for one’s body, and protection of one’s body from unrealistic beauty standards. Each item ranges from 1 (never) to 5 (always). An overall BAS2 score was computed as the mean of all items, with higher scores indicating greater body appreciation. The current study used the Hebrew translation which has been shown to have adequate internal consistency and construct validity [37]. Internal consistency of the BAS2 in this study was McDonald’s *ω* = 0.93.

### Self-Compassion

In order to measure self-compassion, participants completed the Self-Compassion Scale (SCS) [38, 39]. This instrument includes 26-items and assesses the following six aspects of self-compassion: self-kindness, self-judgment, common humanity, isolation, mindfulness, and over-identification. All items were rated on a 5-point scale ranging from 1 (almost never) to 5 (almost always). Negative valance items were reverse coded. An overall SCS score was computed as the mean of all items, with higher scores indicating greater self-compassion. The current study used the Hebrew translation which has been shown to have adequate internal consistency and construct validity [39]. Internal consistency of the SCS in this study was McDonald’s *ω* = 0.94.

### Self-Criticism

Self-criticism was measured using a 23-item subscale of the Depressive Experiences Questionnaire (DEQ-SC) [40]. This subscale reflects concern with failure and with the inability to meet high standards. All items were rated on a 7-point scale ranging from 1 (strongly disagree) to 7 (strongly agree). Scores were obtained by averaging across items, with higher scores indicating greater self-criticism. Internal consistency of the DEQ-SC in this study was McDonald’s *ω* = 0.91.

### Depression

Depression was measured using the 9-item Patient Health Questionnaire (PHQ-9) [41, 42]. All items were rated on a 4-point scale ranging from 0 (not at all) to 3 (nearly every day). Total scores were obtained by summarizing the scores of all items. The total score ranges from 0 to 27 with higher scores indicating higher levels of depression. Internal consistency of the PHQ-9 in this study was McDonald’s *ω* = 0.91.

### Anxiety

Anxiety was measured using the Generalized Anxiety Disorder Scale (GAD-7) [43]. The GAD-7 is a 7-item generalized anxiety measure (panic disorder, social anxiety disorder, and post-traumatic stress disorder). All items were rated on a 4-point scale ranging from 0 (not at all) to 3 (nearly every day). Total scores were obtained by summarizing the scores of all items. The total score ranges from 0 to 21 with higher scores indicating higher levels of anxiety. Internal consistency of the GAD-7 in this study was McDonald’s *ω* = 0.93.

### Statistical Analysis

All statistical analyses were conducted using SPSS 28.0 [44]. Data are described as mean (standard deviation) for continuous data or count (percent) for nominal data. Correlations between study variables were estimated using the Pearson correlation coefficient. Additionally, differences between the study group in demographic or in the main study variables were tested using one-way ANOVA. The moderated serial mediation hypotheses were tested using PROCESS macro, model 91 [45], with 5000 bootstrap samples. For this analysis, the health condition variable (FM or control) was included as a dummy variable. In addition, we included in this analysis all potential covariates that significantly correlated with the outcome variables.

## Results

Participants comprised 146 women with FM and 147 women in the control group. Sample demographics, group differences, and Pearson correlations with the outcome variables are presented in Table 1 [16]. To rule out age and having children as possible confounding variables, the correlations between both variables and depression were calculated for each group separately, yielding non-significant correlations. Group differences in the main study variables and Pearson correlations between the main study variables are presented in Table 2. Pearson correlations between the main study variables for each group separately are presented in Table 3.

**Table 1:** Sample characteristics, group comparison of demographic variables, and Pearson correlations with the study’s outcome variables

Variable	Control (*N* = 147)		FM participants (*N* = 146)			Correlations	
	*M* (SD)	*N* (%)	*M* (SD)	*N* (%)	*F*/*χ*^2^	Depression	Anxiety
Age	30.0 (9.4)		39.7 (12.9)		53.2**	0.14*	0.03
BMI	22.8 (4.8)		25.7 (6.6)		18.7**	0.11	0.07
Has children		35 (17)		99 (49)	50.6**	0.12*	0.07
In a relationship		81 (39)		109 (55)	10.2**	-0.01	0.03

*BMI* body mass index

*p < 0.05, **p < 0.01

**Table 2:** Group differences in and correlations among the main study variables

Variable	Group differences [*M*(SD)]			Correlations			
	Control (*N* = 147)	FM (*N* = 146)	*F*(1, 291)	1	2	3	4
1. Depression	7.4 (5.3)	15.8 (6.0)	161.8**				
2. Anxiety	5.0 (4.7)	10.4 (5.6)	78.8**	0.81**			
3. Body appreciation	3.6 (0.8)	3.2 (0.8)	22.4**	− 0.36**	− 0.34**		
4. Self-compassion	3.2 (0.8)	3.0 (0.8)	7.9**	− 0.42**	− 0.52**	0.64**	
5. Self-criticism	4.1 (1.0)	4.4 (1.0)	6.6*	0.44**	0.55**	− 0.48**	− 0.79**

*p < 0.05; **p < 0.01

**Table 3:** Correlations among the main study variables for the separate groups

Variable	FM group				Control group			
	1	2	3	4	1	2	3	4
1. Depression								
2. Anxiety	0.71**				0.79**			
3. Body appreciation	− 0.19*	− 0.21*			− 0.34**	− 0.32**		
4. Self-compassion	− 0.29**	− 0.46**	0.62**		− 0.53**	− 0.56**	0.63**	
5. Self-criticism	0.41**	0.57**	− 0.42**	− 0.78**	0.49**	0.53**	− 0.51**	− 0.80**

*p < 0.05; **p < 0.01

**Fig. 2:**
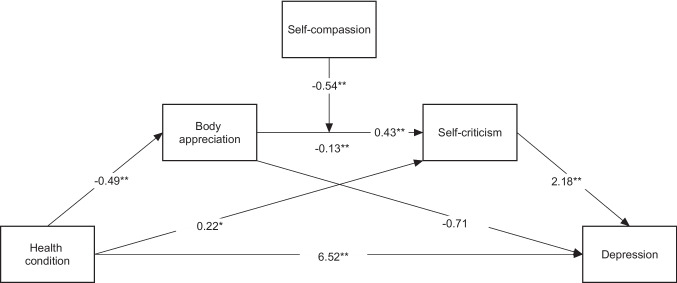
Regression coefficients for moderated serial mediation model using depression as dependent variable.

**Fig. 3:**
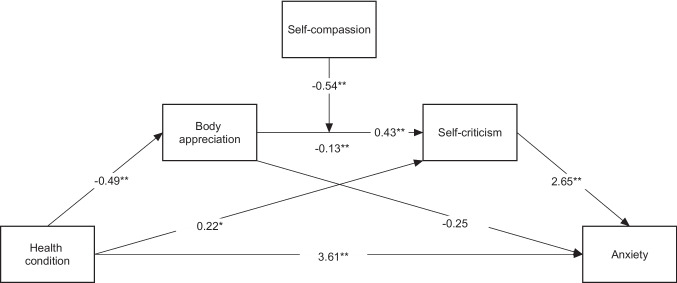
Regression coefficients for moderated serial mediation model using anxiety as dependent variable.

To test the study hypothesis, two moderated serial mediation models were conducted using depression and anxiety levels as dependent variables, separately. The analysis results are presented in Figs. 2 and 3, respectively.

### Depression

Figure 2 shows the results of the moderated mediation analysis model with depression as the outcome variable. A significant direct effect of FM on depression and an indirect effect through self-criticism was obtained (95% CI = [0.11, 0.93]).

A significant moderation effect was found for self-compassion on the relationship between body appreciation and self-criticism (*R*^2^ change = 0.008, *F*(1284) = 7.23, *p* = 0.008). The index of moderated mediation showed a significant moderated mediation effect (index = 0.14, 95% CI = [0.04, 0.30]). The results, shown in Table 4, suggest that the indirect effect is significant only for lower levels of self-compassion.

**Table 4:** Simple effects of the moderated mediation effect of FM on depression through body appreciation and self-criticism. (Simple effects were tested at low (M-SD), medium (M) and high (M + SD) values of self-compassion.)

Moderator value	Depression		Anxiety	
	Effect	95% CI	Effect	95% CI
2.3 (M-SD)	− 0.13	(− 0.32, − 0.01)	− 0.16	(− 0.37, − 0.01)
3.1 (M)	− 0.02	(− 0.16, 0.11)	− 0.02	(− 0.19, 0.13)
3.9 (M + SD)	0.09	(− 0.06, 0.29)	− 0.11	(− 0.08, 0.35)

### Anxiety

Figure 3 shows the results of the moderated mediation analysis model with anxiety as the outcome variable. A significant direct effect of FM on anxiety and an indirect effect through self-criticism was obtained (95% CI = [0.11, 1.07]). The index of moderated mediation showed a significant moderated mediation effect (index = 0.17, 95% CI = [0.04, 0.35]). The results, shown in Table 4, suggest that the indirect effect is significant only for lower levels of self-compassion.

## Discussion

This study aimed to develop an explanatory model for the effect of FM on women’s psychological distress. Specifically, it sought to obtain a deeper understanding of the underlying mechanisms involved in psychological distress among women with FM by identifying the interplay between body appreciation, self-compassion, and self-criticism. In line with previous studies, our results demonstrated higher levels of depression and anxiety in participants with FM than in HP [3]. Furthermore, an examination of the possible mechanism related to the development of psychological distress in women with FM revealed that participants with FM tend to demonstrate lower body appreciation than controls. Lower levels of body appreciation alongside lower levels of self-compassion were related to greater self-criticism and, consequently, higher levels of both depression and anxiety. These findings can be seen to account for the specific challenges facing these women which generate anxiety and distress, such as distrust and non-acceptance of the body, significant functional impairment and disability [46], and overweight and obesity [47]. Moreover, in line with prior theoretical and empirical accounts, they seem to support the shame–self-criticism vicious cycle [17], according to which high levels of shame and self-criticism represent serious disruptions to the capacity for stimulating the inner affiliative systems that are so important for emotion regulation and well-being. Thus, for women with FM, the sick body, which constantly fails to meet societal standards of beauty and function [18], promotes the adoption of a critical and self-deprecating attitude which may, in turn, increase maladaptive defensive responses of distress focused on body image shame [35].

In addition, the fact that lower levels of self-compassion mediated the association between body appreciation and psychological distress might be seen to merely stress the importance of cultivating high levels of self-compassion, as self-compassion enhances improvement in both functional status and well-being among patients with FM [32]. Taken together, the study’s results further demonstrate the possible protective role of self-compassion in indirectly regulating threat-based emotional systems by enabling a more balanced outlook on aversive thoughts and feelings about the body [48].

The current study’s novelty lies in its demonstration of the relationship between body appreciation, self-criticism, self-compassion, and psychological distress—important variables in the compassion-focused therapy theory—all of which are pivotal variables in women with FM. Notably, a systematic review revealed that interventions centered around self-compassion, from online writing exercises to weekly group sessions, had a significant positive impact in reducing psychological distress in those with chronic physical health conditions [49]. Building on this, our model posits that enhancing self-compassion can potentially counteract the detrimental effects of low body appreciation among women with FM. By acknowledging the challenges of living with FM and emphasizing awareness, acceptance, and kindness, clinicians can enhance both distress tolerance and a sense of self-trust in these women [26, 33]. However, when considering our results, a few limitations should be considered. First, the current study used a cross-sectional design, as such, we cannot conclude that FM exacerbates lower body appreciation, greater self-criticism, and psychological distress. Although the diathesis-stress model suggested that people with psychological predisposition, superimposed with the stress of chronic pain, develop depression or anxiety [7, 50]. There is a possibility that psychological distress is linked to self-criticism, which in turn is associated with body appreciation, and then linked to FM. Therefore, our findings should be further validated in a more experimental design, such as longitudinal intervention studies which investigate body image, self-compassion, self-criticism, and psychological distress in women with FM. Second, illness status was reported by the participants and not assessed directly. An additional examination by a medical professional would provide direct information for the association between illness and psychological distress. Third, the study was conducted during the COVID-19 pandemic, which might have influenced the observed results, especially the levels of psychological distress. Fourth, our reliance on the snowball/convenience sample recruitment technique, partly using social media, may have created a sampling bias, according to which individuals who lack access social media were less likely to participate. Finally, the sample comprised only Israeli participants. Considering possible cultural differences in the way people conceptualize illness, it would be beneficial to replicate the findings in diverse cultures and societies.

This study aimed to further investigate the effect of FM on women’s psychological distress by identifying the roles of and interplay between body appreciation, self-criticism, and self-compassion. Based on our results, future studies should further investigate these associations in order to identify potential risk factors and develop tailored psychological interventions for women coping with FM. Future interventions might benefit from addressing body functionality as a possible protective factor against societal pressures [51] which may improve body image and, ultimately, reduce symptoms of depression and anxiety among these patients.

## Supplementary Information

Below is the link to the electronic supplementary material.

Supplementary file1 (DOCX 23 KB)

## Data Availability

The datasets used and analyzed in the current study are available upon reasonable request from the corresponding author.
