# Supplementary material for: Psychological Distress in Women with Fibromyalgia: The Roles of Body Appreciation, Self-Compassion, and Self-Criticism
Source: Int J Behav Med. 2024 Jun 17;32(3):351–9. doi: 10.1007/s12529-024-10302-5 (PMC12134045; doi:10.1007/s12529-024-10302-5)
Supplement: Supplementary file 1 — Supplementary file1 (DOCX 23 KB) [file 12529_2024_10302_MOESM1_ESM.docx]

**Supplementary material**


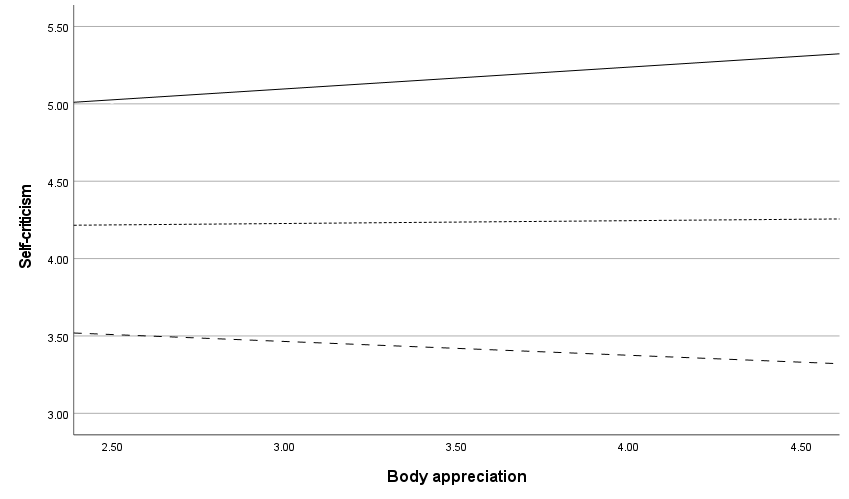


**Figure 4:** Simple effects of the moderation effect of body appreciation on self-criticism, estimated at 3 points of the mediator: M+SD (solid line), M (dotted line) and M-SD (dashed line)
